# Biological mediators involved in tooth resorption following avulsion and delayed replantation: an experimental in vivo study

**DOI:** 10.1590/0103-644020256577

**Published:** 2025-10-24

**Authors:** Léa Assed Bezerra da Silva, Daniele Lucca Longo, Fernanda Maria Machado Pereira Cabral de Oliveira, Júlia Ingryd Targino de Sousa, Kelly Fernanda Molena, Raquel Assed Bezerra da Silva, João Vicente Barbizam, Nestor Cohenca, Maya Fernanda Manfirin Arnez, Marilia Pacifico Lucisano, Francisco Wanderley Garcia de Paula-Silva

**Affiliations:** 1 School of Dentistry of Ribeirão Preto at the University of São Paulo, Ribeirão Preto, SP, Brazil.; 2 University of Washington, Seattle, WA, USA.

**Keywords:** dental trauma, avulsion, tooth replantation, tooth resorption

## Abstract

This study aimed to investigate the biological mediators involved in tooth resorption following permanent tooth avulsion and late replantation at various periods. Premolars of healthy dogs were extracted and divided into: Control Group (CG): negative control, sound teeth (n= 5 teeth / 10 roots); or kept dry and replanted after 20 min (G20): the teeth were replanted after 20 minutes of extraoral time in a dry environment (n= 5 teeth / 10 roots); 60 min (G60): the teeth were replanted after 60 minutes of extraoral time in a dry environment (n= 5); or 90 min (G90): the teeth were replanted after 90 minutes of extraoral time in a dry environment (n= 5 teeth / 10 roots). After this, the teeth were replanted and splinted with 0.4 mm nickel-titanium (Ni-Ti) archwire. After 120 days, the animals were sacrificed, and the tissues were removed for histological processing. The slides were stained for microscopic analysis, submitted to tartrate-resistant acid phosphatase (TRAP) histoenzymology, and immunostained for RANK, RANKL, OPG, alkaline phosphatase, and periostin. Data obtained were submitted to statistical analysis at a 5% significance level. In areas of inflammatory resorption, TRAP + osteoclasts around the replanted teeth were identified, regardless of the duration of extra-alveolar time. RANKL synthesis in this region was more intense after keeping the tooth dry for 90 minutes when compared with other periods (p < 0.05). In areas of replacement resorption, there was less periostin synthesis and a higher level of alkaline phosphatase production (p < 0.05). Late replantation of avulsed teeth resulted in tooth resorption. Inflammatory resorption was characterized by osteoclast recruitment and RANKL synthesis and replacement resorption by alkaline phosphatase synthesis and inhibition of periostin.

## Resumo

Este estudo teve como objetivo investigar as mediadores biológicos envolvidos na reabsorção dentária após avulsão de dente permanente e reimplante tardio em diferentes períodos. Pré-molares de cães saudáveis foram extraídos e divididos em: Grupo Controle (GC) - controle negativo, dentes hígidos (n=5 dentes/10 raízes); ou mantidos secos e reimplantados após 20 min (G20) - dentes reimplantados após 20 minutos de tempo extraoral em ambiente seco (n=5 dentes/10 raízes); 60 min (G60) - dentes reimplantados após 60 minutos de tempo extraoral em ambiente seco (n=5); ou 90 min (G90) - dentes reimplantados após 90 minutos de tempo extraoral em ambiente seco (n=5 dentes/10 raízes). Após isso, os dentes foram reimplantados e esplintados com arco de níquel-titânio (Ni-Ti) de 0,4 mm. Após 120 dias, os animais foram eutanasiados e os tecidos removidos para processamento histológico. As lâminas foram coradas para análise microscópica, submetidas à histoenzimologia para fosfatase ácida tartarato-resistente (TRAP) e imunocoradas para RANK, RANKL, OPG, fosfatase alcalina e periostina. Os dados obtidos foram submetidos à análise estatística com nível de significância de 5%. Em áreas de reabsorção inflamatória, osteoclastos TRAP+ ao redor dos dentes reimplantados foram identificados, independentemente da duração do tempo extra-alveolar. A síntese de RANKL nesta região foi mais intensa após manter o dente seco por 90 minutos quando comparado com outros períodos (p<0,05). Em áreas de reabsorção por substituição, houve menor síntese de periostina e maior produção de fosfatase alcalina (p<0,05). O reimplante tardio de dentes avulsionados resultou em reabsorção dentária. A reabsorção inflamatória foi caracterizada pelo recrutamento de osteoclastos e síntese de RANKL, e a reabsorção por substituição pela síntese de fosfatase alcalina e inibição da periostina.

**Figure:**
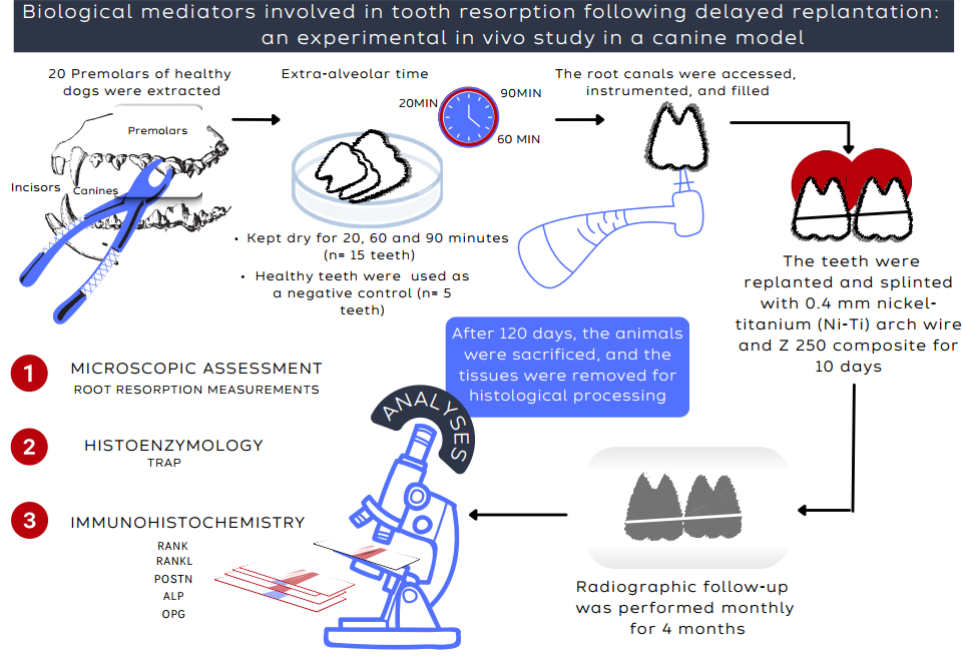

## Introduction

According to the International Association of Dental Traumatology (IADT) ^1^, permanent tooth avulsion represents 0.5 - 16% of all dental injuries and is one of the most serious types. The treatment for dental avulsions is replantation, and the sooner and more appropriately this is performed, the greater the chance of success ^2^ ^,^ ^3^.

Replanted teeth undergo varying degrees of resorption, which are usually asymptomatic, often undiagnosed in clinical examination, and unpredictable ^4^. As microscopic changes occur long before they are noted on radiographs or clinical examination, it is important to know the mechanisms involved in this process. These complications are related to impaired healing of the pulp and periodontal ligament ^3^. If there is minor damage to the periodontal ligament and cementum, a slight osteoclastic activity occurs, and this resorption process may be transient. However, if there is extensive damage to the periodontal ligament and cementum, and the pulp canal becomes infected, inflammatory resorption is likely to occur. When there is an extensive length of extra-alveolar time, and the tooth is stored in a dry environment, root resorption by replacement is the most common complication after replantation of an avulsed tooth. The longer the extra-alveolar time of the avulsed tooth, the lower the potential for healing of the periodontal ligament ^5^. Some studies using cell-based tissue engineering to improve periodontal healing have significantly increased the formation of a new periodontal ligament while reducing adverse outcomes of healing, such as replacement resorption ^6^.

Holding the avulsed tooth by the crown and proper storage until dental replantation is some essential care measures for a good treatment prognosis. Although the most effective storage medium for avulsed teeth cannot be ascertained ^2^, milk, HBSS (Hank's Balanced Salt Solution), saliva, and saline solution can be used to avoid leaving the tooth dry ^1^ ^,^ ^7^.

Mediators that regulate osteogenesis and osteoclastogenesis may be involved in root resorption. The RANK-RANKL-OPG system is considered an important regulator of bone metabolism ^8^. The activator of nuclear receptor kappa factor B (RANK) and its ligand (RANKL) is responsible for the formation and activity of osteoclasts. At the same time, osteoprotegerin (OPG) protects the bone from resorption, acting as a receptor which, by binding to RANKL, prevents it from connecting to RANK ^8^ ^,^ ^9^.

Periostin is expressed by periodontal ligament cells and is involved in wound repair and tissue healing ^10^ ^,^ ^11^ ^,^ ^12^. Alkaline phosphatase (ALP), also a marker of bone formation, is highly expressed in mineralized tissue cells and plays an important role in complex tissue formation ^13^.

Although the IADT emphasized the importance of immediate replantation performed while the patient is still at the accident site or within a short time (less than 15 minutes) so that the periodontal ligament cells remain viable, this does not always occur ^1^. The most frequent cause of tooth avulsion is falls, which can occur in the street, at school, or home ^14^. In those places, parents, caregivers, teachers, and school staff often lack adequate knowledge on how to proceed ^15^. They may need to visit the dental office for replantation, which typically takes longer ^16^.

Therefore, the present study aimed to investigate the biological mediators involved in both replacement and inflammatory tooth resorption following tooth avulsion and late replantation at different periods. This was achieved by simulating the situation that occurs in clinical practice when the patient delays seeking care for hours after a tooth avulsion. Knowledge of the mediators and mechanisms involved in tooth resorption after late replantation would enable the development of more effective treatments, as well as a better understanding of prognosis. The null hypothesis of this study was that extended extra-alveolar dry time before replantation would not significantly alter the expression patterns of biological mediators (RANK/RANKL/OPG, periostin, and alkaline phosphatase) associated with inflammatory and replacement resorption in avulsed teeth.

## Material and methods

The slides used in this section were obtained from the specimen database at the Department of Pediatric Dentistry, School of Dentistry of Ribeirão Preto at the University of São Paulo (FORP-USP), and the material and methods have been detailed in a previous study ^5^.

After approval by the Ethics Committee on Teaching and Research in Animals (007/2012), 4 male Beagle dogs (aged between 12 and 18 months and an average weight of 15 kg) were used. All animal experimentation follows the regulations of CONCEA (Brazil), the AROUCA Law (Law No. 11,794 of October 8, 2008), and is reported following ARRIVE guidelines.

Before the experiment, animals were housed in appropriate spaces with ad libitum access to water and standard dry food. The facility provided segregated areas for experimentation and quarantine in compliance with Brazilian guidelines.

### Surgical procedure

For the anesthesia, the animals received intravenous Neozine (1 mg/kg; Aventis Pharma, Brazil) 15 minutes preoperatively, followed by tiletamine-zolazepam (Zoletil 50, 0.1 ml/kg; Virbac, Brazil) to facilitate endotracheal intubation. General anesthesia was maintained with 6% isoflurane (Abbott Laboratories, Canada) delivered via an inhalation machine (KT-20; Takaoka, Brazil), with continuous monitoring by a veterinarian and intravenous isotonic saline (0.9% NaCl; Glicolabor, Brazil) administration. Parallel radiographs confirmed appropriate root development and absence of apical pathology.

Twenty premolars (n = 40 dental roots) from the maxillary second and third premolars and the mandibular second, third, and fourth premolars (right side) were used. After odontosection, atraumatic root extraction was performed with number 150 sterile forceps (Quinelato, Rio Claro, SP, Brazil), and the canals were instrumented and filled with gutta-percha cones (Dentsply-Herpo, Teresópolis, RJ, Brazil) and AH Plus cement (Dentsply DeTrey, Konstanz, Germany) by the lateral condensation technique. The access cavities were restored with amalgam. Loose debris on the dental root surface was removed by agitating the tooth in saline. The extracted teeth were irrigated and cleaned with sodium chloride 0.9% (physiologic saline water), placed on a sterile gauze pad in a dry environment at room temperature (approximately 25°C) for the designated extra-alveolar periods (20, 60, or 90 minutes) ^5^. All teeth were aseptically manipulated by the crown using sterile surgical gloves and instruments to prevent root surface contamination. Replantation was then performed through controlled digital pressure to ensure proper positioning within the alveolar socket.

The groups were divided according to the period in which the teeth were kept in dry environment before replantation, as follows: Control Group (CG): negative control, sound teeth, not extracted (n= 5 teeth / 10 roots); Group 20 min (G20): the teeth were replanted after 20 minutes of extraoral time in a dry environment (n= 5 teeth / 10 roots); Group 60 min (G60): the teeth were replanted after 60 minutes of extraoral time in dry environment (n= 5); Group 90 min (G90): the teeth were replanted after 90 minutes of extraoral time in a dry environment (n= 5 teeth / 10 roots).

Teeth were splinted with passive retention using a 0.4 mm nickel-titanium (Ni-Ti) archwire and Z 250 composite (3M ESPE, St. Paul, MN, USA) for 10 days. The dogs were maintained on a canned food diet after the surgical procedure. After 120 days, the animals were sacrificed by an anesthetic overdose of 6% sodium pentobarbital, administered intravenously ^5^.

### Microscopic assessment

Blocks of maxillary and mandibular tissue containing the teeth and surrounding alveolar bone were prepared and fixed in 10% formalin. Then, the blocks were washed in running water for 24 hours, decalcified in formic solution (Decal Chemical Corporation, Congers, NY, USA) for 1 week, followed by Immunocal solution (Decal Chemical Corporation, Tallman), NY, USA) for 2 months. Tissues embedded in paraffin were cut into sections with a thickness of 5 µm, with 90 µm intervals parallel to the long axis (mesial-distal-coronal direction) of the roots and stained with hematoxylin-eosin. The slides were examined in an Axiolab 5 Zeiss microscope (Germany) using the Zeiss Zen Lite Blue 3.7 software.

### Histoenzymology for tartrate-resistant acid phosphatase (TRAP)

The presence of osteoclasts was determined by histoenzymatic analysis for TRAP in G20, G60, and G90. Deparaffinized tissue sections were incubated in a solution containing 8 mg of Naphthol Disodium Phosphate AS-MX (Sigma-Aldrich) in 500 μL of NN-dimethylformamide followed by the addition of 50 mL of 0.2 mol/L sodium acetate buffer solution (pH 5.0) containing 70 mg of Fast Red ITR (Sigma-Aldrich). Subsequently, the substrate sodium tartrate dihydrate (50 mmol/L) was added to the solution and incubated at 37°C for two hours. After this, the sections were washed in distilled water and counterstained with Fast Green ^17^. Quantitative analysis of the number of osteoclasts positive for the TRAP enzyme was performed considering the total number of osteoclasts per tooth.

### Immunohistochemistry

Slides were deparaffinized, hydrated in a decreasing series of ethanol (100% to 80%), and maintained in phosphate-buffered saline (PBS). After this, tissue sections were microwaved (7 x 12 seconds at 2-minute intervals) with sodium citrate buffer (pH = 6.0) for antigen recovery. After temperature stabilization, the slides were washed with PBS (3x) for 5 minutes, and endogenous peroxidase activity was blocked with 3% hydrogen peroxide for 40 minutes. Slides were again washed with PBS (3x) for 5 min, and non-specific binding sites were blocked with 5% bovine serum albumin (Sigma-Aldrich) for 60 min.

Tissues were incubated with primary antibodies previously described in our research team ^18^ ^,^ ^19^ to RANK (sc-7626; Santa Cruz Biotechnology, Dallas, TX, USA), RANKL (sc-7628; Santa Cruz Biotechnology), OPG (sc-8468; Santa Cruz Biotechnology), alkaline phosphatase (ab54778; Abcam, Cambridge, MA, USA) and periostin (POSTN) (sc-67233; Santa Cruz Biotechnology Inc.) at 4°C overnight. After this, slides were washed and incubated with biotinylated anti-rabbit and anti-goat secondary antibodies (Biocare Medical, Concord, CA, USA) for one hour, washed in PBS, and incubated using the ABC kit (Avidin Biotinylated complex) for 30 min. The 3,3'-Diaminobenzidine (DAB; Sigma-Aldrich) was used as the enzyme-substrate for 5 min; slides were washed with PBS, counterstained with hematoxylin for 15 s, washed with distilled water, dehydrated in increasing concentrations of ethanol and mounted on Entellan® (Merck, Darmstadt, Germany). Negative controls, in which the primary antibody was omitted, were used to test the specificity of immunostaining. All slides were prepared in the same lot to obtain standardized staining and were evaluated by an experienced, blinded examiner. Scores were attributed to the slides based on staining intensity, categorized as absent, mild, moderate, and intense.

### Statistical analysis

Statistical analyses were performed using GraphPad Prism 6.0. Inflammatory and replacement resorption frequencies (present/absent) in the middle and apical root thirds were compared across groups using chi-square tests (α = 0.05). TRAP-positive osteoclast counts per tooth were analyzed via one-way ANOVA with Tukey’s post hoc test (α = 0.05). Immunohistochemical staining intensity (absent, mild, moderate, or intense) was assessed using the non-parametric Kruskal-Wallis test, followed by Dunn's post hoc test (α = 0.05).

## Results

### Histological Findings

**Figure 1.**
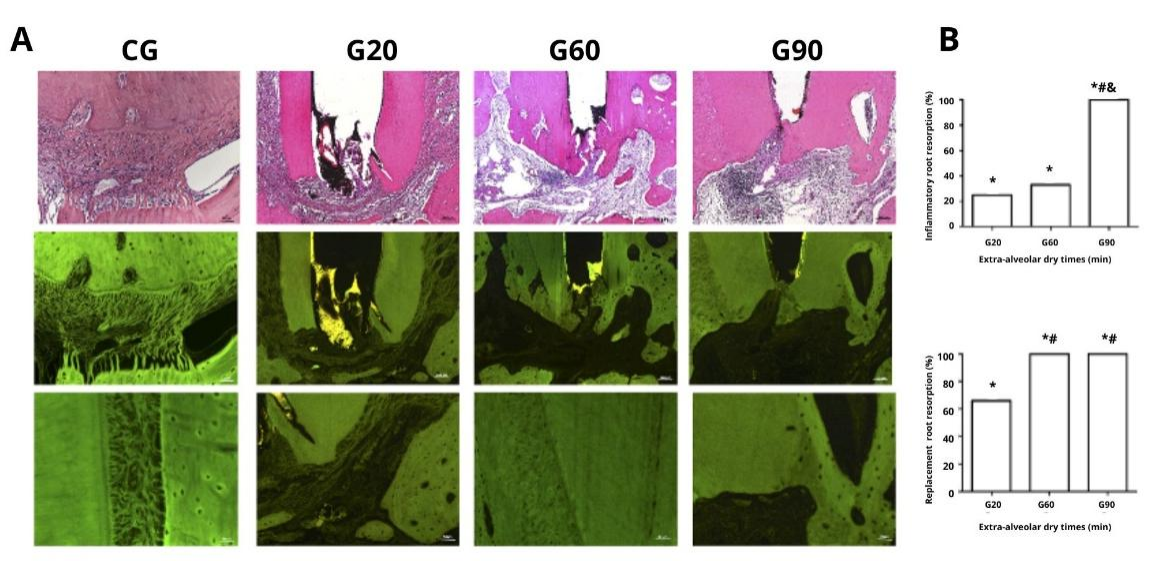
Histopathological analysis of replanted teeth at different extra-alveolar dry times. Panel A shows representative micrographs of the control group (CG) with healthy teeth demonstrating intact periodontal ligament (HE staining at 200× magnification, scale bar = 100 μm; fluorescence microscopy at 200× and 400×, scale bars = 100 μm and 50 μm respectively). The 20-minute group (G20) displays initial inflammatory response (HE staining at 50×, scale bar = 200 μm; fluorescence microscopy at 50× and 100×, scale bars = 200 μm and 100 μm). The 60-minute group (G60) exhibits advanced resorption lacunae (HE staining at 50×, scale bar = 200 μm; fluorescence microscopy at 50× and 400×, scale bars = 200 μm and 50 μm). The 90-minute group (G90) exhibits severe resorption, as indicated by HE staining at 50× (scale bar = 200 μm) and fluorescence microscopy at 50× (scale bar = 200 μm). Panel B quantifies the percentage of inflammatory and replacement resorption across experimental groups (20, 60, and 90 minutes), with the negative control group (CG) showing no resorption. Asterisks indicate statistical significance (*p < 0.05 vs. healthy teeth; #p < 0.05 vs. 20-minute group; &p < 0.05 vs. 60-minute group).

The CG showed no evidence of inflammatory or replacement root resorption. Microscopic examination revealed preserved apical periodontal ligament (PDL) integrity, regular apical cementum with visible cementoblasts, well-organized collagen fibers, and a uniform bone surface with osteoblasts. Fluorescence microscopy further confirmed the presence of healthy tissues and intact collagen fibers (Figure 1A).

Tooth replantation following different dry storage periods demonstrated both inflammatory and replacement resorption patterns (Figure 1A and B). In the G20 group, the apical region exhibited initial inflammatory changes in the periodontal ligament with small resorption areas, while collagen fibers and cementum surface remained essentially regular (Figure 1A). This group showed inflammatory resorption in 25% of specimens and replacement resorption in 66% of cases (Figure 1B).

The G60 group displayed more pronounced pathological changes, including increased PDL space, areas of edema, fibrillar dissociation, and distinct root resorption areas (Figure 1A). The incidence of inflammatory resorption rose to 33%, while replacement resorption affected all specimens (100%) in this group (Figure 1B).

Most severe alterations were observed in G90 specimens, which featured extensive root resorption areas, markedly widened PDL space and intense inflammatory infiltration (Figure 1A). This group demonstrated inflammatory resorption in 100% of cases, representing a significant increase compared to the G20 and G60 groups. While replacement resorption similarly affected all G90 specimens (100%), no statistical difference was observed between G60 and G90 groups for this parameter (Figure 1B).

Comparative analysis revealed that inflammatory resorption was less severe in G20 and G60 groups (without statistical differences between them) and most pronounced in G90. Regarding replacement resorption, G20 showed milder involvement with statistically significant differences from both G60 and G90, though the latter two groups did not differ significantly from each other (Figure 1B).

### TRAP analysis

**Figure 2.**
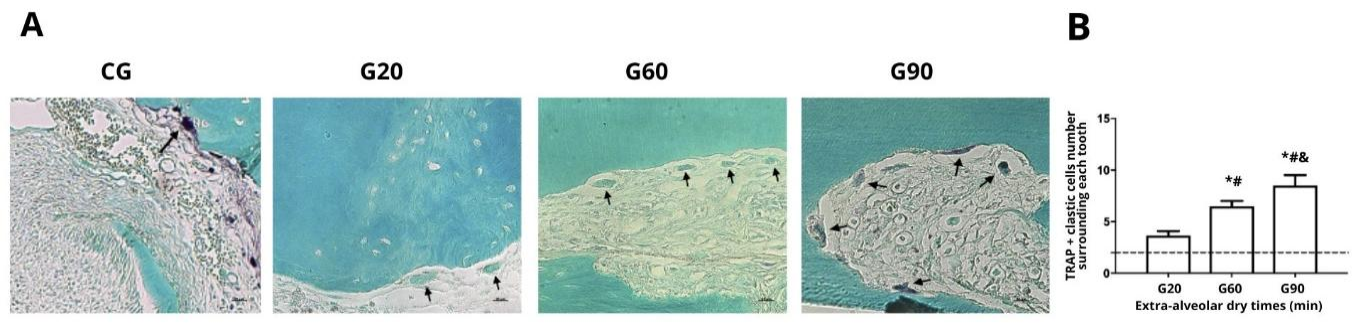
Osteoclast activity analysis by TRAP staining (tartrate-resistant acid phosphatase). Panel A displays TRAP-positive osteoclasts (indicated by arrows) at different extra-alveolar dry periods: healthy control teeth (CG), 20-minute group (G20), 60-minute group (G60), and 90-minute group (G90), all at 100× (scale bar = 20 μm). Panel B presents the quantitative analysis of osteoclast counts, showing a time-dependent increase in TRAP-positive cells across experimental groups. Statistical significance is denoted as follows: *p < 0.05 compared to healthy control teeth (CG), #p < 0.05 compared to the 20-minute group (G20), and &p < 0.05 compared to the 60-minute group (G60). The progressive elevation in osteoclast numbers correlates with extended dry time duration, demonstrating the temporal relationship between extra-alveolar exposure and resorptive activity.

The CG demonstrated the lowest number of TRAP+ osteoclasts (1.5 ± 0.54), significantly fewer than all experimental groups (G20, G60, and G90). Among the experimental groups, G20 showed an intermediate osteoclast count (3.66 ± 1.03), which was substantially lower than both G60 and G90. The G60 group exhibited a marked increase in TRAP+ osteoclasts (6.5 ± 1.22), representing a significant elevation compared to G20, though remaining below G90 levels. The highest osteoclast activity was observed in G90 (8.5 ± 2.50), with counts significantly exceeding all other groups. Statistical analysis confirmed significant differences in TRAP+ osteoclast numbers across all experimental groups (*p* < 0.05) (Figures 2A and 2B). These findings demonstrate an apparent time-dependent increase in osteoclast activity corresponding to extended dry storage periods in replanted teeth.

### Immunohistochemistry

**Figure 3.**
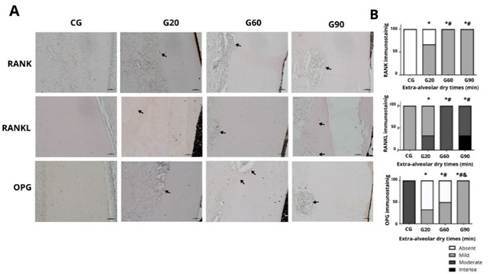
Immunohistochemical analysis of RANK/RANKL/OPG expression. Panel A shows representative micrographs of healthy control teeth (CG) with RANK-negative, RANKL-moderate, and OPG-intense staining; 20-min group (G20) displaying RANK-mild, RANKL-moderate, and OPG-reduced; 60-min group (G60) with RANK-moderate, RANKL-intense, and OPG-weak staining; 90-min group (G90) showing RANK-strong, RANKL-intense, and OPG-minimal expression, all 20× (scale bar = 100 μm). Panel B quantifies staining intensity (*p<0.05 vs. CG; #p<0.05 vs. G20; &p<0.05 vs. G60), demonstrating the progressive RANKL↑/OPG↓ imbalance during delayed replantation.

Immunohistochemical analysis revealed distinct expression patterns of biological markers across different tissue compartments (pulp chamber, dentin, periodontal ligament, and bone), correlating with varying degrees of inflammatory and replacement resorption (Figure 3A and B). The CG exhibited absent RANK expression, mild RANKL immunostaining, and intense OPG presence. Comparative analysis of experimental groups demonstrated progressive changes in these immunomarkers: group G20 showed absent-to-mild RANK, moderate-to-mild RANKL, and absent-to-mild OPG expression, while group G60 presented mild RANK, moderate-to-mild RANKL, and absent OPG (Figure 3B). Group G90 demonstrated mild RANK expression, intense-to-mild RANKL, and mild OPG (Figure 3B).

Statistical analysis revealed significant intergroup differences. For RANK expression, CG differed significantly from all experimental groups (G20, G60, and G90), with G60 and G90 showing the most pronounced changes (p<0.05) but no significant difference between them. Both RANKL and OPG expression demonstrated statistically significant differences (p<0.05) among all groups in pairwise comparisons, indicating progressively altered RANKL/OPG ratios corresponding to extended extraoral dry times (Figure 3 B).

**Figure 4.**
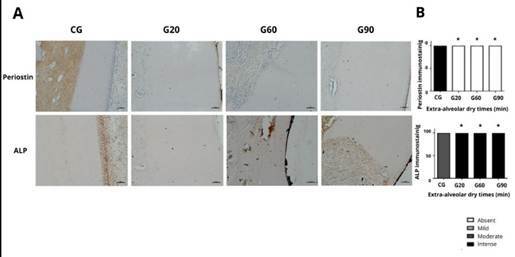
Immunohistochemical analysis of periostin and alkaline phosphatase (ALP) expression patterns. Panel A displays representative photomicrographs of healthy control teeth (CG) showing intense periostin expression and moderate ALP activity; 20-minute group (G20) with absent periostin and intense ALP; 60-minute group (G60) demonstrating absent periostin and intense ALP; and 90-minute group (G90) showing absent periostin and intense ALP, all 20× (scale bar = 100 μm). Panel B presents a quantitative analysis of staining intensity, revealing complete periostin loss in all experimental groups (*p < 0.05 vs. CG) and sustained high ALP expression throughout the 20-, 60-, and 90-minute dry periods, indicating persistent mineralization activity despite progressive periodontal damage.

Further evaluation of periostin and ALP expression patterns (Figure 4) showed markedly different profiles between CG and G20, G60, and G90 (*p* < 0.05). While CG specimens displayed intense periostin immunostaining, this marker was completely absent in all experimental groups (G20, G60, and G90). Conversely, ALP expression showed moderate intensity in controls but markedly increased to intense levels across all experimental groups. These findings collectively demonstrate significant alterations in key biological markers associated with tooth resorption processes following different durations of dry storage prior to replantation.

## Discussion

The present study provides comprehensive evidence that delayed replantation following avulsion initiates a time-dependent cascade of periodontal damage, with distinct pathological mechanisms emerging at different dry storage intervals. Our experimental model, which evaluated dry periods of 20, 60, and 90 minutes, mirrors the clinical reality where immediate replantation within the IADT's recommended 15-minute window ^1^ is frequently unattainable due to logistical constraints, patient anxiety, and limited emergency preparedness among bystanders ^20^. The progressive increase in both inflammatory and replacement resorption frequencies across our experimental groups - from 25% inflammatory resorption at 20 minutes to 100% incidence of both types at 90 minutes - offers quantitative validation of clinical observations that extra-alveolar duration critically determines outcomes.

Our findings reveal two parallel yet interconnected pathways that drive resorption. The RANK-RANKL-OPG system showed progressive dysregulation, with RANKL expression intensifying from mild (20 min) to intense (90 min), while OPG remained depressed throughout. This pattern of increasing RANKL coupled with decreasing OPG following prolonged drying highlights the PDL's vulnerability and reinforces the concept that extended extra-alveolar durations accelerate root resorption ^8^ ^,^ ^9^ ^,^ ^19^. The imbalance directly correlated with escalating osteoclast activity (TRAP+ cells: 3.66±1.03 at 20 min vs. 8.5±2.50 at 90 min), aligning with studies demonstrating RANKL's pivotal role in osteoclastogenesis ^21^ ^,^ ^22^ while establishing its time-dependent progression post-avulsion. Critically, the near-absence of OPG's protective effect suggests rapid system failure during dry storage, creating permissive conditions for inflammatory and replacement resorption ^8^ ^,^ ^9^.

Simultaneously, the absence of periostin in experimental groups, in contrast to its consistent presence in controls, highlights its critical role in maintaining PDL integrity as an essential mediator of PDL homeostasis and wound healing ^10^ ^,^ ^11^ ^,^ ^12^. Periostin's rapid disappearance following avulsion likely creates permissive conditions for ankylosis. This effect was particularly pronounced in the 60- and 90-minute groups, where replacement resorption reached 100% incidence. The concurrent elevation of alkaline phosphatase at all time points suggests that attempted osseous repair occurs alongside progressive root resorption, potentially explaining the clinically observed progression of replacement resorption even in asymptomatic cases ^13^.

The 60-minute threshold proved particularly consequential in our study, marking the point at which replacement resorption became universal and RANKL-mediated inflammatory responses intensified significantly. This finding corroborates epidemiological data showing teeth replanted beyond this window have 59-85.7% ankylosis rates ^23^ ^,^ ^24^ while providing a mechanistic explanation for these clinical observations. Notably, our immunohistochemical results suggest that mediator changes preceding visible resorption may serve as early warning indicators, with RANKL upregulation and periostin loss potentially predicting adverse outcomes before radiographic changes become apparent.

Several clinical factors may interact with these biological processes. While our controlled experiment focused exclusively on dry time, clinical outcomes are undoubtedly influenced by additional variables, including patient age, root development stage, and concomitant injuries ^1^ ^,^ ^14^. The frequent delays in seeking treatment - often due to inadequate public knowledge about proper tooth storage and transportation ^15^ ^,^ ^16^ exacerbate these biological challenges, making our experimental model highly relevant to real-world scenarios where teeth are frequently stored improperly for extended periods.

Our study design intentionally prioritized mediator analysis over clinical simulation, which explains confident methodological choices. The use of endodontically treated teeth eliminated confounding factors from pulpal infection, allowing for the isolation of periodontal healing dynamics. While this approach enhances internal validity ^5^ ^,^ ^17^ ^,^ ^21^, it may underestimate resorption rates in clinical cases where combined endodontic-periodontal involvement is common ^23^. Similarly, the standardized 120-day observation period provides robust intermediate-term data but cannot capture longer-term progression patterns that might influence clinical decision-making.

Potential limitations require thoughtful consideration. In our study, we evaluated replantation after extended extra-alveolar periods exceeding 20 min ^20^. Future studies could incorporate an additional control group assessing the IADT guideline of 15 minutes or less for optimal tooth preservation ^1^. The use of polyclonal antibodies developed for human or rodent targets in a canine model, although common in translational research, raises potential questions about cross-reactivity. Nevertheless, the high evolutionary conservation of the RANK/RANKL/OPG pathway and periostin among mammalian species underscores the biological significance of our findings, which align with previous studies investigating inflammatory conditions in dogs ^18^ ^,^ ^19^. The exclusion of cervical root regions from analysis, while methodologically necessary to avoid extraction artifacts, means we cannot comment on resorption patterns in this clinically important zone. Future studies incorporating more extended observation periods and species-specific reagents could further strengthen these observations.

These findings carry important implications for clinical practice and research. The demonstration that mediators' changes commence within 20 minutes underscores the critical importance of public education programs emphasizing immediate replantation ^1^ ^,^ ^25^ or proper wet storage ^2^. Our results suggest potential therapeutic opportunities targeting the identified mediators' pathways through localized RANKL inhibition or periostin supplementation strategies. The consistent ALP elevation across all experimental groups raises intriguing questions about whether modulating mineralization processes could slow the progression of replacement resorption.

In conclusion, this study provides a comprehensive timeline of mediators' events underlying post-avulsion resorption, bridging the gap between clinical guidelines and biological reality. Our findings confirm that dry storage duration determines resorption severity through distinct but interconnected pathways involving RANKL-mediated inflammation and periostin-dependent healing failure. While immediate replantation remains the gold standard, understanding these mechanisms opens new avenues for improving outcomes when treatment delays are inevitable. Future research should explore whether mediation interventions targeting these pathways could extend the viability window for avulsed teeth.
